# 4-[(1*E*,3*E*,5*E*)-6-(4-Pyrid­yl)hexa-1,3,5-trien­yl]pyridine

**DOI:** 10.1107/S1600536809029092

**Published:** 2009-07-29

**Authors:** Mamoun M. Bader

**Affiliations:** a Department of Chemistry, Pennsylvania State University at Hazleton, 76 University Drive, Hazleton, PA 18202, USA

## Abstract

The two independent mol­ecules in the asymmetric unit of the title compound, C_16_H_14_N_2_, are planar [dihedral angle between the terminal pyridine rings  = 1.76 (2)°] and each display an all-*trans* configuration of C=C double bonds. One of the two mol­ecules lies about a center of inversion. The dihedral angle between the two pyridine rings in the mol­ecule lying on a general position is 1.65 (2)°.

## Related literature

For acceptor-terminated polyenes, see: Gao *et al.* (2004 ▶). For the synthesis, see: Woitellier *et al.* (1989 ▶). For a related structure, see: Pham (2009 ▶).
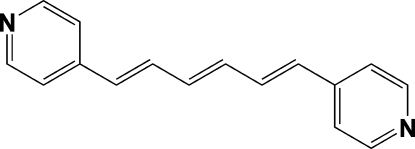


## Experimental

### 

#### Crystal data

C_16_H_14_N_2_
*M* *_r_* = 234.29
Monoclinic, 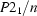

*a* = 5.837 (1) Å
*b* = 17.171 (4) Å
*c* = 19.227 (4) Å
β = 97.685 (4)°
*V* = 1909.8 (7) Å^3^
*Z* = 6
Mo *K*α radiation
μ = 0.07 mm^−1^
*T* = 173 K
0.44 × 0.24 × 0.22 mm

#### Data collection

Bruker SMART Platform CCD diffractometer
Absorption correction: none
18771 measured reflections
3366 independent reflections
2460 reflections with *I* > 2σ(*I*)
*R*_int_ = 0.028

#### Refinement

*R*[*F* ^2^ > 2σ(*F* ^2^)] = 0.032
*wR*(*F* ^2^) = 0.089
*S* = 1.01
3366 reflections
328 parameters
All H-atom parameters refined
Δρ_max_ = 0.09 e Å^−3^
Δρ_min_ = −0.15 e Å^−3^

### 

Data collection: *SMART* (Bruker, 2001 ▶); cell refinement: *SAINT* (Bruker, 2001 ▶); data reduction: *SAINT*; program(s) used to solve structure: *SIR2002* (Burla *et al.*, 2003 ▶); program(s) used to refine structure: *SHELXL97* (Sheldrick, 2008 ▶); molecular graphics: *SHELXTL* (Sheldrick, 2008 ▶); software used to prepare material for publication: *SHELXL97*.

## Supplementary Material

Crystal structure: contains datablocks I, global. DOI: 10.1107/S1600536809029092/ng2615sup1.cif

Structure factors: contains datablocks I. DOI: 10.1107/S1600536809029092/ng2615Isup2.hkl

Additional supplementary materials:  crystallographic information; 3D view; checkCIF report

## Figures and Tables

**Fig. 1.**
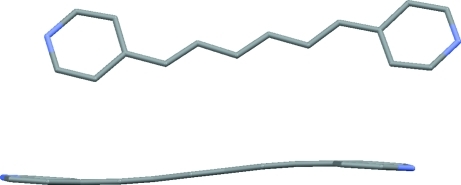
Ellipsoid plot

**Fig. 2.**
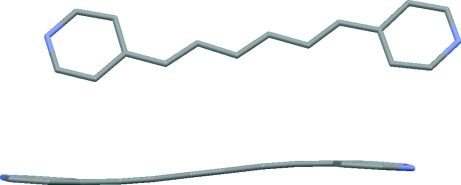
4-((1E,3E,5E)-6-(pyridin-4-yl)hexa-1,3,5-trienyl)pyridine.

**Fig. 3.**
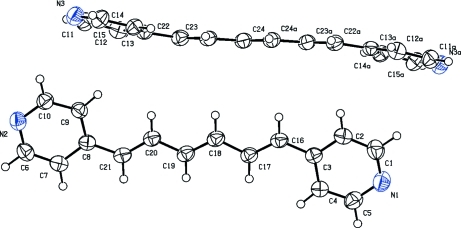
Crystal packing viewed along the a axis.

## supplementary crystallographic information

### Comment

Pyridyl-terminated polyenes have been investigated for studying electron reactions with applications in biology, inorganic reaction mechanisms and molecular electronics. They have also been used in the synthesis of coordination polymers as well as template for solid state reactions.

For related systems of acceptor terminated polyenes, see: Gao *et al.* (2004); Pham (2009). For literature related to the synthesis, see: Woitellier *et al.* (1989).

### Experimental

Synthesis was carried out following literature procedures (Woitellier *et al.*,  1989) as follows: a solution of potassium *tert*-butoxide (1 2. l g in 200 ml of glyme) was added dropwise to a solution of tetraethyl ((*E*)-2-butene-1,4-diyl)diphosphonate (16.4 g, 0.05 mol) and pyridine-4-carboxaldehyde (10.70 g, 0.10 mol) in 100 ml of glyme at room temperature. After the addition of the base was complete, the resulting mixture was stirred at room temperature for 16 h. Cold water (*ca* 300–400 ml) was added and the product isolated by vacuum filtration (3.5 g, 30% crude yield) and recrystallized from acetone (2.3 g, 19%): mp 196–197. Crystals were grown from DMF.

### Refinement

All hydrogen atoms were found from the difference map and refined with individual isotropic displacement parameters.

### Crystal data

**Table:**

C_16_H_14_N_2_	*F*(000) = 744
*M**_r_* = 234.29	*D*_x_ = 1.222 Mg m^−^^3^
Monoclinic, *P*2_1_/*n*	Mo *K*α radiation, λ = 0.71073 Å
Hall symbol: -P 2yn	Cell parameters from 867 reflections
*a* = 5.837 (1) Å	θ = 2.5–27.5°
*b* = 17.171 (4) Å	µ = 0.07 mm^−^^1^
*c* = 19.227 (4) Å	*T* = 173 K
β = 97.685 (4)°	Block, yellow
*V* = 1909.8 (7) Å^3^	0.44 × 0.24 × 0.22 mm
*Z* = 6	

### Data collection

**Table:**

Bruker SMART Platform CCD diffractometer	2460 reflections with *I* > 2σ(*I*)
Radiation source: normal-focus sealed tube	*R*_int_ = 0.028
graphite	θ_max_ = 25.0°, θ_min_ = 1.6°
ω scans	*h* = −6→6
18771 measured reflections	*k* = −20→20
3366 independent reflections	*l* = −22→22

### Refinement

**Table:**

Refinement on *F*^2^	Primary atom site location: structure-invariant direct methods
Least-squares matrix: full	Secondary atom site location: difference Fourier map
*R*[*F*^2^ > 2σ(*F*^2^)] = 0.032	Hydrogen site location: inferred from neighbouring sites
*wR*(*F*^2^) = 0.089	All H-atom parameters refined
*S* = 1.01	*w* = 1/[σ^2^(*F*_o_^2^) + (0.048*P*)^2^ + 0.1535*P*] where *P* = (*F*_o_^2^ + 2*F*_c_^2^)/3
3366 reflections	(Δ/σ)_max_ = 0.001
328 parameters	Δρ_max_ = 0.09 e Å^−^^3^
0 restraints	Δρ_min_ = −0.15 e Å^−^^3^

### Special details

**Table:**

Geometry. All e.s.d.'s (except the e.s.d. in the dihedral angle between two l.s. planes) are estimated using the full covariance matrix. The cell e.s.d.'s are taken into account individually in the estimation of e.s.d.'s in distances, angles and torsion angles; correlations between e.s.d.'s in cell parameters are only used when they are defined by crystal symmetry. An approximate (isotropic) treatment of cell e.s.d.'s is used for estimating e.s.d.'s involving l.s. planes.
Refinement. Refinement of *F*^2^ against ALL reflections. The weighted *R*-factor *wR* and goodness of fit *S* are based on *F*^2^, conventional *R*-factors *R* are based on *F*, with *F* set to zero for negative *F*^2^. The threshold expression of *F*^2^ > σ(*F*^2^) is used only for calculating *R*-factors(gt) *etc*. and is not relevant to the choice of reflections for refinement. *R*-factors based on *F*^2^ are statistically about twice as large as those based on *F*, and *R*- factors based on ALL data will be even larger.

### Fractional atomic coordinates and isotropic or equivalent isotropic displacement parameters (Å^2^)

**Table:**

	*x*	*y*	*z*	*U*_iso_*/*U*_eq_	
N1	0.5720 (2)	0.58755 (7)	−0.07734 (6)	0.0569 (3)	
N2	−0.45576 (19)	1.10512 (6)	0.36976 (5)	0.0481 (3)	
N3	−0.00550 (19)	1.24674 (6)	0.23495 (6)	0.0497 (3)	
C1	0.6991 (3)	0.65228 (9)	−0.06772 (8)	0.0543 (4)	
H1	0.838 (3)	0.6547 (8)	−0.0931 (7)	0.062 (4)*	
C2	0.6494 (2)	0.71361 (8)	−0.02588 (7)	0.0462 (3)	
H2	0.751 (2)	0.7581 (8)	−0.0194 (7)	0.052 (4)*	
C3	0.4553 (2)	0.71081 (7)	0.00886 (6)	0.0388 (3)	
C4	0.3222 (2)	0.64283 (7)	−0.00011 (7)	0.0431 (3)	
H4	0.185 (2)	0.6363 (7)	0.0227 (6)	0.044 (3)*	
C5	0.3870 (3)	0.58440 (8)	−0.04271 (7)	0.0511 (4)	
H5	0.293 (2)	0.5370 (8)	−0.0495 (6)	0.056 (4)*	
C6	−0.5830 (2)	1.04076 (8)	0.35549 (7)	0.0479 (3)	
H6	−0.722 (3)	1.0359 (8)	0.3800 (7)	0.060 (4)*	
C7	−0.5305 (2)	0.98292 (8)	0.31014 (7)	0.0445 (3)	
H7	−0.628 (2)	0.9374 (8)	0.3018 (6)	0.049 (4)*	
C8	−0.3348 (2)	0.98987 (7)	0.27589 (6)	0.0385 (3)	
C9	−0.2039 (2)	1.05746 (7)	0.29001 (6)	0.0411 (3)	
H9	−0.064 (2)	1.0657 (7)	0.2688 (6)	0.041 (3)*	
C10	−0.2692 (2)	1.11169 (8)	0.33646 (7)	0.0449 (3)	
H10	−0.176 (2)	1.1575 (7)	0.3472 (6)	0.044 (3)*	
C11	−0.1535 (2)	1.23391 (8)	0.17687 (7)	0.0487 (3)	
H11	−0.298 (2)	1.2632 (7)	0.1723 (7)	0.055 (4)*	
C12	−0.1104 (2)	1.18348 (8)	0.12416 (7)	0.0453 (3)	
H12	−0.223 (2)	1.1766 (8)	0.0831 (7)	0.057 (4)*	
C13	0.0985 (2)	1.14333 (7)	0.12916 (6)	0.0384 (3)	
C14	0.2518 (2)	1.15541 (7)	0.19037 (7)	0.0415 (3)	
H14	0.401 (2)	1.1294 (7)	0.1986 (6)	0.049 (4)*	
C15	0.1931 (2)	1.20645 (8)	0.24030 (7)	0.0473 (3)	
H15	0.300 (2)	1.2158 (8)	0.2839 (7)	0.059 (4)*	
C16	0.3959 (2)	0.77741 (7)	0.05039 (6)	0.0414 (3)	
H16	0.504 (2)	0.8205 (7)	0.0528 (6)	0.040 (3)*	
C17	0.2082 (2)	0.78441 (7)	0.08307 (6)	0.0404 (3)	
H17	0.100 (2)	0.7412 (7)	0.0822 (6)	0.043 (3)*	
C18	0.1516 (2)	0.85257 (7)	0.12091 (6)	0.0432 (3)	
H18	0.260 (2)	0.8965 (7)	0.1235 (6)	0.049 (4)*	
C19	−0.0351 (2)	0.85901 (7)	0.15427 (7)	0.0431 (3)	
H19	−0.140 (2)	0.8147 (7)	0.1538 (6)	0.047 (4)*	
C20	−0.0919 (2)	0.92526 (7)	0.19440 (6)	0.0414 (3)	
H20	0.012 (2)	0.9683 (8)	0.1966 (6)	0.044 (3)*	
C21	−0.2736 (2)	0.92738 (7)	0.23017 (6)	0.0420 (3)	
H21	−0.376 (2)	0.8825 (8)	0.2281 (6)	0.049 (4)*	
C22	0.1515 (2)	1.09529 (7)	0.07030 (7)	0.0420 (3)	
H22	0.030 (2)	1.0935 (7)	0.0295 (7)	0.050 (4)*	
C23	0.3498 (2)	1.05781 (7)	0.06546 (7)	0.0417 (3)	
H23	0.473 (2)	1.0581 (7)	0.1052 (6)	0.044 (3)*	
C24	0.3997 (2)	1.01778 (7)	0.00320 (7)	0.0439 (3)	
H24	0.275 (2)	1.0192 (7)	−0.0377 (7)	0.052 (4)*	

### Atomic displacement parameters (Å^2^)

**Table:**

	*U*^11^	*U*^22^	*U*^33^	*U*^12^	*U*^13^	*U*^23^
N1	0.0517 (7)	0.0549 (8)	0.0644 (8)	0.0045 (6)	0.0091 (6)	−0.0115 (6)
N2	0.0471 (7)	0.0512 (7)	0.0468 (6)	0.0063 (5)	0.0095 (5)	0.0009 (5)
N3	0.0557 (7)	0.0448 (6)	0.0486 (7)	0.0072 (5)	0.0073 (6)	−0.0015 (5)
C1	0.0454 (9)	0.0607 (9)	0.0581 (9)	0.0038 (7)	0.0118 (7)	−0.0031 (7)
C2	0.0441 (8)	0.0453 (8)	0.0494 (8)	−0.0044 (6)	0.0065 (6)	0.0033 (6)
C3	0.0428 (7)	0.0382 (7)	0.0340 (6)	0.0018 (5)	0.0002 (5)	0.0060 (5)
C4	0.0428 (8)	0.0420 (7)	0.0441 (7)	−0.0017 (6)	0.0049 (6)	0.0013 (6)
C5	0.0499 (9)	0.0437 (8)	0.0587 (9)	−0.0027 (6)	0.0034 (7)	−0.0047 (7)
C6	0.0413 (8)	0.0560 (9)	0.0477 (8)	0.0050 (6)	0.0107 (6)	0.0079 (7)
C7	0.0410 (8)	0.0444 (8)	0.0479 (8)	−0.0026 (6)	0.0052 (6)	0.0071 (6)
C8	0.0396 (7)	0.0377 (7)	0.0371 (7)	0.0021 (5)	0.0012 (5)	0.0065 (5)
C9	0.0384 (7)	0.0425 (7)	0.0431 (7)	−0.0015 (6)	0.0076 (6)	0.0014 (6)
C10	0.0451 (8)	0.0422 (8)	0.0473 (8)	−0.0008 (6)	0.0060 (6)	−0.0024 (6)
C11	0.0467 (8)	0.0480 (8)	0.0518 (8)	0.0099 (6)	0.0080 (7)	0.0049 (6)
C12	0.0437 (8)	0.0490 (8)	0.0417 (8)	0.0016 (6)	−0.0003 (6)	0.0050 (6)
C13	0.0437 (7)	0.0339 (6)	0.0377 (7)	−0.0030 (5)	0.0060 (6)	0.0042 (5)
C14	0.0411 (8)	0.0376 (7)	0.0450 (7)	0.0022 (6)	0.0027 (6)	−0.0003 (6)
C15	0.0531 (9)	0.0423 (7)	0.0445 (8)	0.0033 (6)	−0.0009 (6)	−0.0040 (6)
C16	0.0497 (8)	0.0359 (7)	0.0379 (7)	−0.0048 (6)	0.0029 (6)	0.0035 (5)
C17	0.0504 (8)	0.0361 (7)	0.0340 (7)	−0.0020 (6)	0.0027 (6)	0.0031 (5)
C18	0.0553 (9)	0.0364 (7)	0.0370 (7)	−0.0019 (6)	0.0026 (6)	0.0023 (6)
C19	0.0517 (8)	0.0369 (7)	0.0394 (7)	−0.0006 (6)	0.0011 (6)	0.0013 (6)
C20	0.0476 (8)	0.0364 (7)	0.0391 (7)	−0.0025 (6)	0.0022 (6)	0.0018 (5)
C21	0.0463 (8)	0.0356 (7)	0.0431 (7)	−0.0027 (6)	0.0022 (6)	0.0030 (6)
C22	0.0480 (8)	0.0395 (7)	0.0377 (7)	−0.0033 (6)	0.0028 (6)	0.0017 (5)
C23	0.0503 (8)	0.0351 (7)	0.0394 (7)	−0.0046 (6)	0.0049 (6)	0.0004 (5)
C24	0.0541 (8)	0.0363 (7)	0.0415 (7)	−0.0046 (6)	0.0073 (7)	0.0006 (6)

### Geometric parameters (Å, °)

**Table:**

N1—C1	1.3355 (18)	C11—H11	0.975 (14)
N1—C5	1.3436 (18)	C12—C13	1.3931 (18)
N2—C6	1.3394 (17)	C12—H12	0.964 (14)
N2—C10	1.3396 (16)	C13—C14	1.3952 (17)
N3—C11	1.3354 (17)	C13—C22	1.4663 (17)
N3—C15	1.3422 (17)	C14—C15	1.3765 (18)
C1—C2	1.380 (2)	C14—H14	0.971 (13)
C1—H1	0.999 (15)	C15—H15	0.989 (13)
C2—C3	1.3910 (18)	C16—C17	1.3394 (18)
C2—H2	0.964 (13)	C16—H16	0.969 (12)
C3—C4	1.3999 (18)	C17—C18	1.4395 (18)
C3—C16	1.4627 (18)	C17—H17	0.973 (13)
C4—C5	1.3796 (19)	C18—C19	1.3408 (19)
C4—H4	0.970 (13)	C18—H18	0.982 (13)
C5—H5	0.980 (14)	C19—C20	1.4382 (18)
C6—C7	1.3830 (19)	C19—H19	0.976 (13)
C6—H6	0.997 (14)	C20—C21	1.3390 (18)
C7—C8	1.3974 (18)	C20—H20	0.954 (13)
C7—H7	0.969 (13)	C21—H21	0.973 (13)
C8—C9	1.3961 (17)	C22—C23	1.3380 (18)
C8—C21	1.4616 (17)	C22—H22	0.985 (13)
C9—C10	1.3788 (18)	C23—C24	1.4436 (18)
C9—H9	0.969 (12)	C23—H23	0.978 (12)
C10—H10	0.964 (13)	C24—C24^i^	1.341 (3)
C11—C12	1.3815 (18)	C24—H24	0.997 (13)
			
C1—N1—C5	115.69 (12)	C13—C12—H12	119.3 (8)
C6—N2—C10	115.81 (12)	C12—C13—C14	116.18 (12)
C11—N3—C15	115.75 (12)	C12—C13—C22	119.88 (11)
N1—C1—C2	124.04 (14)	C14—C13—C22	123.85 (12)
N1—C1—H1	116.0 (8)	C15—C14—C13	119.46 (12)
C2—C1—H1	120.0 (8)	C15—C14—H14	118.7 (7)
C1—C2—C3	120.14 (13)	C13—C14—H14	121.9 (7)
C1—C2—H2	120.4 (8)	N3—C15—C14	124.58 (13)
C3—C2—H2	119.5 (8)	N3—C15—H15	115.0 (8)
C2—C3—C4	116.35 (12)	C14—C15—H15	120.4 (8)
C2—C3—C16	120.18 (12)	C17—C16—C3	126.49 (12)
C4—C3—C16	123.45 (12)	C17—C16—H16	118.4 (7)
C5—C4—C3	119.21 (13)	C3—C16—H16	115.1 (7)
C5—C4—H4	119.6 (7)	C16—C17—C18	124.57 (13)
C3—C4—H4	121.2 (7)	C16—C17—H17	119.5 (7)
N1—C5—C4	124.55 (13)	C18—C17—H17	115.9 (7)
N1—C5—H5	116.1 (8)	C19—C18—C17	124.39 (13)
C4—C5—H5	119.3 (8)	C19—C18—H18	118.2 (7)
N2—C6—C7	123.92 (13)	C17—C18—H18	117.4 (7)
N2—C6—H6	116.0 (8)	C18—C19—C20	125.63 (13)
C7—C6—H6	120.1 (8)	C18—C19—H19	118.8 (7)
C6—C7—C8	119.99 (13)	C20—C19—H19	115.5 (8)
C6—C7—H7	120.5 (8)	C21—C20—C19	123.54 (13)
C8—C7—H7	119.5 (8)	C21—C20—H20	119.9 (7)
C9—C8—C7	116.10 (12)	C19—C20—H20	116.5 (7)
C9—C8—C21	123.76 (12)	C20—C21—C8	127.09 (12)
C7—C8—C21	120.12 (12)	C20—C21—H21	118.8 (7)
C10—C9—C8	119.73 (12)	C8—C21—H21	114.1 (8)
C10—C9—H9	119.7 (7)	C23—C22—C13	126.71 (12)
C8—C9—H9	120.5 (7)	C23—C22—H22	117.8 (7)
N2—C10—C9	124.44 (13)	C13—C22—H22	115.3 (7)
N2—C10—H10	115.9 (7)	C22—C23—C24	124.00 (13)
C9—C10—H10	119.6 (7)	C22—C23—H23	119.9 (7)
N3—C11—C12	123.79 (13)	C24—C23—H23	116.1 (7)
N3—C11—H11	116.4 (8)	C24^i^—C24—C23	124.67 (17)
C12—C11—H11	119.8 (8)	C24^i^—C24—H24	119.5 (8)
C11—C12—C13	120.21 (13)	C23—C24—H24	115.8 (8)
C11—C12—H12	120.5 (8)		
			
C5—N1—C1—C2	−0.5 (2)	C11—C12—C13—C22	−174.59 (11)
N1—C1—C2—C3	−0.7 (2)	C12—C13—C14—C15	−1.72 (18)
C1—C2—C3—C4	1.43 (18)	C22—C13—C14—C15	174.85 (12)
C1—C2—C3—C16	−176.92 (12)	C11—N3—C15—C14	1.1 (2)
C2—C3—C4—C5	−1.03 (18)	C13—C14—C15—N3	0.1 (2)
C16—C3—C4—C5	177.26 (11)	C2—C3—C16—C17	175.93 (12)
C1—N1—C5—C4	0.9 (2)	C4—C3—C16—C17	−2.3 (2)
C3—C4—C5—N1	−0.1 (2)	C3—C16—C17—C18	−177.16 (11)
C10—N2—C6—C7	0.60 (19)	C16—C17—C18—C19	−179.16 (12)
N2—C6—C7—C8	−0.2 (2)	C17—C18—C19—C20	177.79 (11)
C6—C7—C8—C9	−0.64 (17)	C18—C19—C20—C21	−176.06 (13)
C6—C7—C8—C21	177.48 (11)	C19—C20—C21—C8	175.54 (11)
C7—C8—C9—C10	1.12 (17)	C9—C8—C21—C20	−1.5 (2)
C21—C8—C9—C10	−176.93 (11)	C7—C8—C21—C20	−179.51 (12)
C6—N2—C10—C9	−0.08 (19)	C12—C13—C22—C23	175.18 (13)
C8—C9—C10—N2	−0.8 (2)	C14—C13—C22—C23	−1.3 (2)
C15—N3—C11—C12	−0.6 (2)	C13—C22—C23—C24	−173.53 (11)
N3—C11—C12—C13	−1.0 (2)	C22—C23—C24—C24^i^	177.66 (15)
C11—C12—C13—C14	2.12 (18)		

Symmetry codes: (i) −*x*+1, −*y*+2, −*z*.
